# Bayesian Inference Under the Multispecies Coalescent with Ancient DNA Sequences

**DOI:** 10.1093/sysbio/syae047

**Published:** 2024-07-30

**Authors:** Anna A Nagel, Tomáš Flouri, Ziheng Yang, Bruce Rannala

**Affiliations:** Department of Evolution and Ecology, University of California, 1 Shields Avenue, Davis, CA 95616, USA; Department of Genetics, Evolution, and Environment, University College London, Gower Street, London WC1E 6BT, UK; Department of Genetics, Evolution, and Environment, University College London, Gower Street, London WC1E 6BT, UK; Department of Evolution and Ecology, University of California, 1 Shields Avenue, Davis, CA 95616, USA

**Keywords:** aDNA, BPP, multispecies coalescent, tip dating

## Abstract

Ancient DNA (aDNA) is increasingly being used to investigate questions such as the phylogenetic relationships and divergence times of extant and extinct species. If aDNA samples are sufficiently old, expected branch lengths (in units of nucleotide substitutions) are reduced relative to contemporary samples. This can be accounted for by incorporating sample ages into phylogenetic analyses. Existing methods that use tip (sample) dates infer gene trees rather than species trees, which can lead to incorrect or biased inferences of the species tree. Methods using a multispecies coalescent (MSC) model overcome these issues. We developed an MSC model with tip dates and implemented it in the program BPP. The method performed well for a range of biologically realistic scenarios, estimating calibrated divergence times and mutation rates precisely. Simulations suggest that estimation precision can be best improved by prioritizing sampling of many loci and more ancient samples. Incorrectly treating ancient samples as contemporary in analyzing simulated data, mimicking a common practice of empirical analyses, led to large systematic biases in model parameters, including divergence times. Two genomic datasets of mammoths and elephants were analyzed, demonstrating the method’s empirical utility.

Ancient DNA (aDNA) sequences are increasingly available for many species due to advances in sequencing technology. Whole genome sequences from aDNA exist for several groups of extinct species, including neanderthals (Green et al., 2010), woolly and Columbian mammoths (Palkopoulou et al., 2015, 2018), woolly rhinoceros (Lord et al., 2020), and cave bears (Fortes et al., 2016). Genome sequences from aDNA also exist for many extant species, for example humans (Rasmussen et al., 2010; Nielsen et al., 2017) and maize (Ramos-Madrigal et al., 2016). More limited aDNA data are available for an even wider variety of species such as bison (Soubrier et al., 2016), polar bears (Miller et al., 2012), pigs (Horsburgh et al., 2022), and many plants and pathogens (Orlando et al., 2021). These data have opened the door to new ways to investigate long-standing questions in phylogenetics and population genetics, such as phylogenetic relationships between extinct and extant species, their divergence times, and their demographic and migration histories.

A key feature that distinguishes aDNA from modern DNA is the (potentially large) differences in ages among sampled aDNA sequences; in conventional studies of modern DNA all samples are contemporary. The importance of accounting for the sampling date of non-contemporary sequences has long been recognized for viral sequences, in particular RNA viruses (Drummond et al., 2003). Due to the high substitution rates of RNA viruses, substitutions may occur in lineages that have not yet been sampled during the intervals between sampling events, creating differences in expected branch lengths between lineages descended from a common ancestor, even under a strict molecular clock. With molecular sequence data, the amount of evolution observed is determined by the product of substitution rate and time. Sequences sampled atdifferent times may have detectable differences in expected substitutions if either the mutation rate is high (as with viral data) or the time interval between sampling events is large (as with older aDNA samples). Similar to fossil calibrations, sampling dates provide information about substitution rates, allowing absolute divergence times (e.g., days or years) and absolute substitution rates to be jointly estimated (Li et al., 1988; Rambaut, 2000).

Whole genomes of aDNA contain much information for detecting even small differences of expected numbers of substitutions; one might speculate that increasing the number of loci will improve estimates of parameters such as absolute divergence times and mutation rate even with younger samples because each locus is an independent source of information. As more loci are added, the expected difference in branch lengths between lineages sampled at different times is more precisely estimated, thus improving estimates of both mutation rate and absolute divergence times. An advantage of dating with aDNA samples over fossil calibrations is that the position of the sample in the phylogeny can potentially be inferred from the sequence data whereas fossils must be assigned to ancestral nodes based solely on sparse morphological characters and are probably frequently misassigned.

Another reason to develop statistical models for analyzing aDNA is the potential for biased estimates if sample dates are ignored. Several studies have analyzed aDNA by treating all samples (including aDNA) as contemporary (Rohland et al., 2010; Palkopoulou et al., 2018). This should lead to underestimation of divergence times. It is poorly understood how great the absolute time interval between samples must be before it affects inference when sampling dates are not explicitly modeled.

## Analyses of aDNA Without Tip Dates

Population samples of aDNA have been analyzed using several methods which do not explicitly use sampling dates. Two of these, pairwise sequential Markovian coalescent (PSMC) (Li and Durbin, 2011) and coalHMM, (Mailund et al., 2012) are commonly used methods for inferring ancestral demography (past effective population size through time) based on an approximation to the coalescent process with recombination. However, both allow inference for small samples (e.g., two sequences from one diploid individual in the case of PSMC). In order to estimate population sizes in continuous time with time in calendar units, mutation rate and generation time are treated as known in PSMC, though both are uncertain. When two or more individuals have been sampled that share an ancestral population, researchers have used PSMC independently on the samples and then aligned the demographic histories inferred with PSMC to determine when the populations diverged. This is problematic because data from different individuals are analyzed independently and divergence times are not estimated directly.

When multiple sequences are sampled from multiple species, multispecies coalescent (MSC) models in MCMCcoal (an early version of BPP) have been used to infer divergence times and effective population sizes with aDNA, with the ancient sequences treated as if they were contemporary (Rohland et al., 2010). The effect of ignoring sample ages for programs such as coalHMM and MCMCcoal should depend on the time period spanned by the sampling dates of the sequences relative to the divergence times of the populations but is in general unknown.

## Analyses of aDNA With Tip Dates

The program BEAST is used to analyze data from multiple species to estimate divergence times, accommodating dated tips (Suchard et al., 2018; Bouckaert et al., 2019). BEAST does not employ the MSC and ignores the difference between gene trees and the species tree. Using divergence times for different clades in gene trees as an estimate of the species divergence time (e.g., Chang et al., 2017) leads to overestimation of species divergence times since the common ancestor of a gene must be older than the common ancestor of the species (Gillespie and Langley, 1979; Angelis and Dos Reis, 2015). The MSC with dated tips is available in the package StarBeast3 in BEAST2 (Douglas et al., 2022) for estimating divergence times, effective population sizes and mutation rate. However, StarBeast3 assumes that all sequences from any particular species are sampled at the same time.

## Prospects for MSC Analysis of aDNA

Phylogenetic methods based on the multispecies coalescent (MSC), such as BPP and StarBeast3, provide a more realistic model to analyze sequence data from multiple species or populations. These methods can estimate divergence times and effective population sizes and a variety of migration and hybridization histories. The BPP program allows analyses of datasets of thousands of loci, multiple individuals per population and multiple populations (or species) (Flouri et al., 2018, 2023). Moreover, the methods are statistically consistent and make complete use of all information available in the data.

Here, we describe an MSC model with tip dates that allows any number of distinct sampling times within each population (or species) assuming a fixed population (species) tree. We implement this model in the Bayesian phylogenetic inference program BPP. We assess the performance of the method using simulations under a variety of population histories and investigate the impact of incorrectly treating ancient sequences as contemporary. We apply the new method to analyze two elephant and mammoth nuclear DNA and mtDNA datasets.

## Materials and Methods

### Theory: Overview of the MSC Model with Tip Dating

**Table 1:** Four different time scales produce the same inference

Time unit	Coalescent rate	Mutation rate	Heterozygosity θ	Likelihood L⁢(d)
(i) Year	1/2⁢N⁢g	μ	2×2⁢N⁢g×μ=θ	L⁢(Δ⁢y×μ)
(ii) Generation	1/2⁢N	$\mu_{g}=\mu \,g$	$2\times 2\,N\times \mu_{g}=\theta$	$L\,\left(\Delta \,y/g\times \mu_{g}\right)$
(iii) Coalescent time unit				
(2⁢N generations)	1	$2\,N\,\mu_{g}$	$2\times 1\times 2\,N\,\mu_{g}=\theta$	$L\,\left(\Delta \,y/2\,N\,g\times 2\,N\,\mu_{g}\right)$
(iv) Mutational time unit	2/θ	1	2×θ/2×1=θ	L⁢(Δ⁢y⁢μ×1)

Note: Heterozygosity is twice the coalescent waiting time (which is the reciprocal of the coalescent rate) times the mutation rate. Likelihood is for two sequences sampled at $y_{1}$ and $y_{2}$ years before present (ybp), which coalesce at year y with the separation time to be $\Delta \,y=y-y_{1}+y-y_{2}$ years, and the sequence distance is defined as the product of separation time times the mutation rate. For example with the coalescent time unit (which is 2⁢N generations), the coalescent rate for a sequence pair is 1 per time unit, the mutation rate is 2⁢N⁢μ⁢g mutations per site per time unit, and the separation time between the two sequences is Δ⁢y/2⁢N⁢g time units.

The standard MSC model assumes that all sequences are sampled at the present time. We modify the MSC to allow a joint analysis of ancient and modern samples. We assume a fixed species tree topology with no gene flow. We also assume that each sample can be assigned *a priori* to a population which represents a tip on the species tree, and no sequences are sampled from ancestral populations (which correspond to internal nodes on the species tree). We consider diploid species, so that there are 2⁢N sequences at any locus in a population of size N. For a haploid system, our 2⁢N should be replacedby N.

Let $\,X=\left\{\,x_{i}\right\}$ be the sequence data with $\,x_{i}$ to be the alignment of sequences at locus i including the sampling times. Let $\,G=\left\{G_{i}\right\}$ be the gene trees, where $G_{i}$ is the gene tree at locus i and includes both the topology and coalescent times (node ages). Let g be the generation time, in years per generation (Takahata et al., 1995). Let the mutation rate be μ per site per year or $\mu_{g}$ per site per generation, with $\mu_{g}=\mu \,g$.

With sampling times for sequences, we may choose to use different time scales. Here we use the case of two sequences sampled from one population of size N (with heterozygosity θ=4⁢N⁢g⁢μ) to illustrate that the use of different time scales produces equivalent inference (Table 1). Suppose the two sequences are sampled at times $y_{1}$ and $y_{2}$ (years before present or ybp), with $y_{1}<y_{2}$. The gene tree in this case is the coalescent time between the two sequences. In Table 1, we summarized four time scales: (i) calendar time (with one time unit to be a year, say; other units such as a day may be used similarly), (ii) generation, (iii) the coalescent time unit of 2⁢N generations, and (iv) the mutational time scale (with one time unit to be the expected amount of time taken to accumulate one mutation per site).

Consider the calendar time or ybp (Table 1(i)), and let the coalescent time be $y>y_{2}$ ybp. This has theprobability


$$f\,\left(y|N,g\right)\,d\,y=\frac{1}{2\,N}\,e^{\left(-1/2\,N\right)\,\left(y-y_{2}\right)/g}d\,y/g,y>y_{2}.$$ (1)

The likelihood for the sequence data, L⁢(d), depends on the distance $d=\left(y-y_{1}+y-y_{2}\right)\,\mu$. Thus the joint conditional distribution of the coalescent time y and the parameters in the model (N,g,μ) is


$$\begin{array}{cc} & f\,\left(N,g,\mu ,y|\,X\right)\propto f\,\left(y|N,g\right)\,L\,\left(d\right)\\ & =\frac{1}{2\,N\,g}\,e^{\left(-1/\left(2\,N\,g\right)\right)\,\left(y-y_{2}\right)}\times L\,\left(\left(y-y_{1}+y-y_{2}\right)\,\mu \right),y>y_{2}. \end{array}$$ (2)

One may use $\Theta_{\text{i}}=\left(N\,g,\mu \right)$ as the set of identifiable parameters, as N and g are confounded.

Next, suppose we use the mutational time scale, with one time unit to be the expected time to accumulate one mutation per site. The coalescent time t=y⁢μ is measured in mutations per site. The joint conditional then becomes


$$\begin{array}{cc} & f\,\left(\theta ,\mu ,t|\,X\right)\propto f\,\left(t|\theta ,\mu \right)\,L\,\left(d\right)\\ & =\frac{2}{\theta }\,e^{\left(-2/\theta \right)\,\left(t-y_{2}\,\mu \right)}\times L\,\left(t-y_{1}\,\mu +t-y_{2}\,\mu \right),t>y_{2}\,\mu . \end{array}$$ (3)

The set of parameters may be defined as $\Theta_{\text{iv}}=\left(\theta ,\mu \right)$.

The two formulations (as well as ii and iii in Table 1) produce the same inference, as long as the priors are compatible. Note that $\Theta_{\text{i}}$ and $\Theta_{\text{iv}}$ constitute a one-to-one mapping or reparametrization, whereas MCMC algorithms such as implemented in BPP sample gene trees (i.e., node age y in years in equation 2 or t in mutations in equation 3) as well as parameters, integrating out y from equation 2 and t from equation 3 result in the same likelihood function for the parameters.

In this paper, we use the mutational time scale, measuring time by the number of mutations per site. Note that the generation time g does not need to be specified unless one wants to explicitly estimate N. Similarly, population divergence time in the MSC (τ in BPP) is measured in units of expected mutations and the definition does not require knowledge of g. Let Θ be the vector of parameters of the species tree, Θ=(⁢τ,⁢θ), where ⁢τ is the vector of speciation times and ⁢θ is the vector of mutation scaled effective population sizes, both measured in expected number of mutations. For example, speciation time in ybp is given as $\tau^{△}=\tau /\mu$.

The joint posterior probability of the divergence times, effective population sizes, and gene trees is given by


f⁢(Θ,⁢G,μ|⁢X)∝f⁢(⁢G|Θ)⋅P⁢(⁢X|⁢G,μ)⋅f⁢(μ,Θ) (4)

The phylogenetic likelihood P⁢(⁢X|⁢G,μ) is calculated under the JC model assuming a strict molecular clock (Felsenstein, 1981). The gene tree density given the population divergence times (τs), the population sizes (θs), and the sampling times, f⁢(⁢G|Θ), is given by combining the coalescent model with serial samples of Rodrigo and Felsenstein (1999) and MSC model of Rannala and Yang (2003).

**Figure 1:**
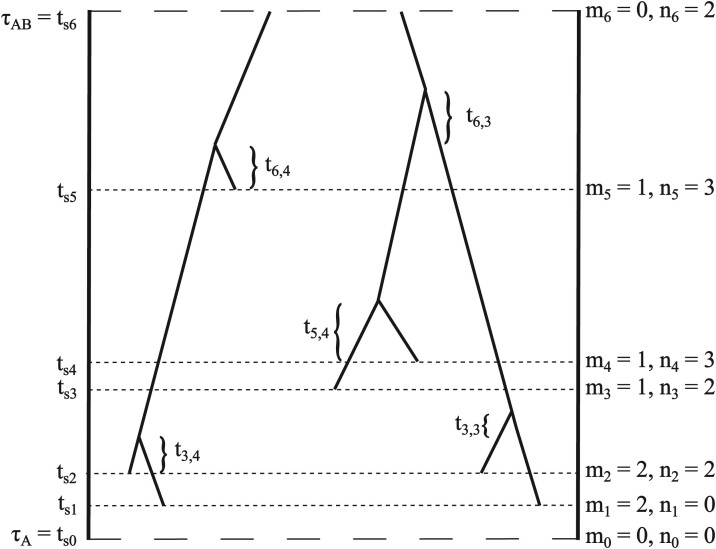
Part of a gene tree in population A. Samples for population A are taken at five distinct times, $t_{s\,1}<t_{s\,2}<\cdots <t_{s\,5}$, with time running backwards. The five sampling times split the time period for population A, ($\tau_{A},\tau_{A\,B}$), into 6 epochs during which no samples are added and the number of lineages can only decrease due to coalescence. For convenience, we let $t_{s\,0}=\tau_{A}$ be the starting time and $t_{s\,6}=\tau_{A\,B}$ the ending time for population A. The number of lineages existing at $t_{s\,i}$ equals the number of lineages sampled ($m_{i}$) plus the number surviving to $t_{s\,i}$ ($n_{i}$). For example, 3 lineages survive to time $t_{s\,5}$, so $n_{5}=3$, and one lineage is sampled at time $t_{s\,5}$, so $m_{5}=1$. Waiting times until coalescent events are written with two subscripts. The first indexes the epoch and the second the number of lineages before the coalescent event. For example, during epoch $\left(t_{s\,5},t_{s\,6}\right)$ the waiting time until the first coalescent event is $t_{6,4}$, where the 6 refers to the sixth epoch and the 4 refers to coalescent event that reduces the number of lineages from 4 to 3.

The gene tree density is a product over populations. For each population, we use the sampling times to split the time duration for the population into epochs (time intervals) within which no new samples are added and the number of lineages can only decrease (Fig. 1). Let there be E sampling epochs. The sampling times in expected number of substitutions are $t_{s\,1}<t_{s\,2}<\ldots <t_{s\,\left(E-1\right)}<t_{s\,E}$, with $t_{s\,i}=y_{s\,i}\,\mu$ where $y_{s\,i}$ is the sample time in ybp. For convenience, we also let $t_{s\,0}$ be the starting time (either time present or population divergence time) and $t_{s\,\left(E+1\right)}$ the ending time for the population. At time $t_{s\,i}$, $m_{i}$ sequences are sampled, with $m_{0}=0$. Let the number of lineages surviving to time $t_{s\,i}$ be denoted $n_{i}$. Let the waiting time for the coalescent event which reduces the number of lineages from k to k−1 during epoch i be denoted $t_{i,k}$ (Fig. 1). For an epoch i with no coalescent events, there will not be any defined $t_{i,j}$. The probability density of the gene tree for one population is


$$\begin{array}{cc} f\,\left(\,G|\Theta ,\mu \right) & =\prod_{i=1}^{E+1}(.\prod_{j=n_{i}+1}^{n_{i-1}+m_{i-1}}\left[\frac{2}{\theta }\exp\left\{-\frac{j\,\left(j-1\right)}{\theta }t_{i,j}\right\}\right]\\ & \times \exp\{-\frac{n_{i}\,\left(n_{i}-1\right)}{\theta }(t_{s\,i}-[t_{s\,\left(i-1\right)}\\ & +\sum_{j=n_{i}+1}^{n_{i-1}+m_{i-1}}t_{i,j}])\}.). \end{array}$$ (5)

The root population does not have a time $t_{s\,\left(E+1\right)}$. The density for the root population is


$$\begin{array}{cc} f\,\left(\,G|\Theta ,\mu \right) & =\prod_{i=1}^{E}(\prod_{j=n_{i}+1}^{n_{i-1}+m_{i-1}}\left[\frac{2}{\theta }\exp\left\{-\frac{j\,\left(j-1\right)}{\theta }t_{i,j}\right\}\right]\\ & \times \exp\{-\frac{n_{i}\,\left(n_{i}-1\right)}{\theta }(t_{s\,i}-[t_{s\,\left(i-1\right)}\\ & +\sum_{j=n_{i}+1}^{n_{i-1}+m_{i-1}}t_{i,j}])\})\\ & \times \prod_{j=2}^{n_{E}+m_{E}}\left[\frac{2}{\theta }\exp\left\{-\frac{j\,\left(j-1\right)}{\theta }t_{\left(E+1\right),j}\right\}\right]. \end{array}$$ (6)

The density for the complete gene tree at every locus is given by multiplying across populations.

### The MCMC Algorithms

We implemented the MSC model with dated tips in the Bayesian inference program BPP. Markov chain Monte Carlo (MCMC) is used to sample from the joint conditional distribution of the gene trees and parameters. Here we describe new and modified MCMC proposals.

#### Updating substitution rate (μ)

The sample times are specified by the user in units of calendar time before present. They are fixed during the MCMC. The calendar times are multiplied by μ to become expected number of substitutions, as all of the calculations in BPP are in these units. Currently in BPP, internally branch lengths are stored in expected number of substitutions, and previously BPP did not have time calibration capabilities. Therefore, times in expected number of substitutions are used because it required substantially less modifications to the program. When a proposal changes μ, all sample times (in units of substitutions) must be updated to preserve the absolute sample times.


$$t_{s\,i}^{*}=t_{s\,i}\times \frac{\mu^{*}}{\mu },$$ (7)

where the superscript * indicates a proposed value. This ensures the absolute sample times are constant. Since each sample is assigned to a population, the divergence times impose constraints on the possible values of μ. Change in μ must not move the sample between populations. More specifically,


$$y_{s\,i}\times \mu^{*}=t_{s\,i}^{*}<\tau$$ (8)

This gives a local upper bound for $\mu^{*}$ as $\min\left\{\tau /y_{s\,i}\right\}$ for all samples in a population. The minimum of this bound over all loci for all populations gives the global upper bound used in the proposal. The lower bound is an arbitrarily small positive number. We propose a new substitution rate, $\mu^{*}$, on a log scale with sliding window, reflecting at the bounds (Yang, 2014, p. 221–226)


$$\mu^{*}=\mu \times c=\mu \times e^{\epsilon \,x},$$ (9)

where ϵ is the fine-tune parameter (or step size) and x is a random variable drawn from a Bactrian Laplace distribution (Yang and Rodríguez, 2013). This move has a proposal ratio of c (Yang, 2014, p. 225). The tip dates in units of expected substitutions undergo a transformation given by


$$t_{s\,i}^{*}=t_{s\,i}\times \frac{\mu^{*}}{\mu }=y_{s\,i}\times \mu^{*}$$ (10)

To solve for the proposal ratio, the Jacobian is calculated.


$$\frac{\partial t_{s\,i}}{\partial t_{s\,i}^{*}}=\left|\frac{\mu }{\mu^{*}}\right|.$$ (11)

The reverse move is the inverse so the proposal ratio is one.

**Figure 2:**
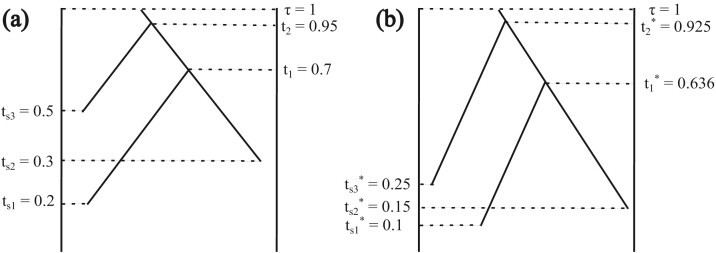
Part of a gene tree within a population (**a**) before and (**b**) after a new substitution rate is proposed. Here $\mu /\mu^{*}=2$ and the tip dates are updated to be: $b_{1}=\max\left(t_{s\,1},t_{s\,2}\right)=0.3$, $b_{1}^{*}=\max\left(t_{s\,1}^{*},t_{s\,2}^{*}\right)=0.15$, $b_{2}=\max\left(t_{s\,1},t_{s\,2},t_{s\,3}\right)=0.5$, and $b_{2}^{*}=\max\left(t_{s\,1}^{*},t_{s\,2}^{*},t_{s\,3}^{*}\right)=0.25.$ Not drawn to scale.


Updating tip ages in units of expected number of substitutions without updating the coalescent times can lead to the coalescent times being younger than their daughter nodes, which is not allowed. This type of move could be rejected, but rejection leads to poor mixing. To improve mixing of the MCMC, we jointly update the coalescent times in the populations when updating tip dates. Let $b_{i}$ be the age (in expected number of substitutions) of the oldest sample that is descendant from a node i in the gene tree. We keep the age of $t_{i}$ relative to $b_{i}$ and τ constant (Fig. 2).

$$\frac{\tau -t_{i}^{*}}{\tau -b_{i}^{*}}=\frac{\tau -t_{i}}{\tau -b_{i}}$$ (12)

Let $h_{i}=\left(\tau -t_{i}\right)/\left(\tau -b_{i}\right)$ and rearranging the equation,


$$t_{i}^{*}=\tau -h_{i}\times \left(\tau -b_{i}^{*}\right)$$ (13)

To derive the proposal ratio,


$$J\,\left(h\right)=\det\frac{\partial \left(t_{1},t_{2},\ldots ,t_{n}\right)}{\partial \left(h_{1},h_{2},\ldots ,h_{n}\right)}=\prod_{i=1}^{n}\left(\tau -b_{i}^{*}\right)$$ (14)

Proposing the change to μ on a log scale has a proposal ratio of c. The proposal ratio for the move is thus


$$c\times \frac{J\,\left(h^{*}\right)}{J\,\left(h\right)}=c\times \prod_{i=1}^{n}\frac{\tau -b_{i}^{*}}{\tau -b_{i}}$$ (15)

It is possible for this move to propose times such that a daughter node is older than a parent node in the gene tree. In this case, the move is rejected.

For example, consider the gene tree embedded in the species tree of Fig. 2. The sample time or coalescent time, in expected number of substitutions, is labeled for each node. A new value of μ is proposed using equation 9. The sample times ($t_{s\,1},t_{s\,2},t_{s\,3}$) are updated using equation 7. Then the coalescent times ($t_{1},t_{2}$) are updated using equation 13, resulting in the gene tree in Fig. 2b.

#### Updating species divergence times

The speciation times, τ, are proposed so that the sample times bound the possible node ages. The age of a node is constrained above by the age of the parent node, $\tau_{u}$, and below by the oldest daughter node $\tau_{l}$. Samples cannot change populations, imposing an additional constraint on speciation times. For a given population, $t_{s\,E}$ is the oldest sample across all loci. Since the samples only occur in tip populations of the species tree, $\tau_{l}=0\leq t_{s\,E}$. The speciation time for the parent population is thus bounded below by $t_{s\,E}$. As in the previous implementation, a proposed move that is outside of the bounds is reflected to be within bounds.

#### Gene tree SPR

**Figure 3:**
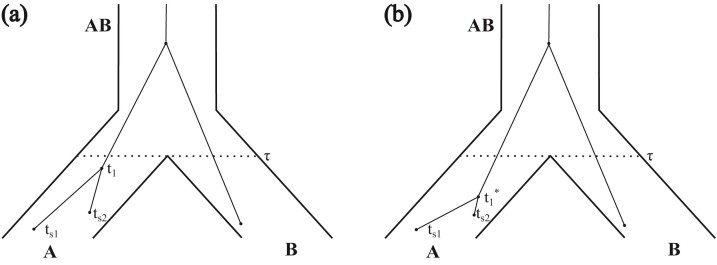
A gene tree to illustrate the gene-tree SPR move. If the sample at time $t_{s\,1}$ is pruned, the lower bound on the reattachment time is $t_{s\,1}$ since there are nodes in population A that are not in the subtree. If the reattachment time it less than $t_{s\,2}$, there is no possible reattachment point so the move is rejected. If the sample at time $t_{s\,2}$ is pruned instead, the lower bound on reattachment is $t_{s\,2}$. Since $t_{s\,1}$ is younger than $t_{s\,2}$, it will always be possible to attach the sample at time $t_{s\,2}$ at the proposed time. If the sample from population B is pruned instead, the lower bound on the reattachment time is τ since there are no other nodes within population B.

The subtree-pruning-and-regrafting (SPR) proposal applied to gene trees (Rannala and Yang, 2017) is modified to allow for dated samples. In the implementation without sample dates, a node or subtree in the gene tree is selected to be pruned. The branch between the node and the parent node is removed. To choose a time to reattach the subtree, a bound on the youngest possible reattachment time is found. If the population in which the node exists has nodes that are not part of the subtree, the bound is equal to the node age of the pruned node. If the population does not have nodes which are not part of the subtree, the bound is the speciation time for the youngest ancestral population which has gene tree nodes that are not part of the subtree (Fig. 3). The upper bound is an arbitrarily large number. A reattachment time is proposed and reflected at the bounds.

With dated tips, it is possible that a population will have gene-tree nodes that are not part of the subtree, but are older than the proposed time. This may occur when the pruned node is younger than all samples that are not part of the subtree (Fig. 3). In this case, the move is rejected. Rejection due to this constraint can occur only with the youngest sample in a population and does not affect most proposals, having little impact on mixing, and is used for simplicity.

As an example, consider the gene tree and species tree in Fig. 3a. If the node sampled at time $t_{s\,1}$ is pruned, the lower bound on reattachment is $t_{s\,1}$. It is possible to propose a time between $t_{s\,1}$ and $t_{s\,2}$. In this case, the move is rejected as there are no branches on which to attach in this time interval. If the node sampled at time $t_{s\,2}$ is pruned, the lower bound is $t_{t\,2}$, and there will always be at least one branch (leading to the node at $t_{s\,1}$) on which to attach. The node in population B could also be pruned. The lower bound for attachment is τ, as there are no other nodes in population B. Similarly, the node at time $t_{1}$ could be pruned and have a lower bound for attachment of τ. In Fig. 3b, the node at time $t_{s\,1}$ is pruned, and a time $t_{1}^{*}$ is proposed for reattachment. In this case, the topology of the gene tree did not change. If $t_{1}^{*}$ were older than τ, the node could also have been grafted to the branch from the node inpopulation B.

#### Other proposals

The proposals to the gene tree coalescent times and the proposal on θ did not require modifications. The mixing proposal, which multiplies all times or node ages by a scale factor and divides all rates by the same factor so that the likelihood does not change (Thorne et al., 1998), is turned off in the current implementation. Traditional mixing proposals that do not change the likelihood are not possible with tip dating because the gene tree branch lengths cannot all be proportionally rescaled while fixing the tip dates in real time.

#### Validation of the implementation

To test our inference method, we modified the simulation method in BPP to accommodate serial sampling as described in SI Section 1. We have extensively tested our simulation and MCMC implementations. Each MCMC proposal was tested by running under the prior, which is equivalent to setting the likelihood of the data to one. The MCMC results were compared against the analytical results for the prior distributions when these were known. However, the tip dates impose constraints on τs and μ, changing their prior distribution so that the ‘effective’ priors used by the algorithm differ from the user-specified gamma prior. This is similar to the situation in Bayesian relaxed-clock dating where the effective priors on divergence times differ from user-specified fossil-calibration densities (Rannala, 2016). In our tests, we used rejection simulation to determine the effective prior.

An independent simulation program was written to sample from the effective prior for a four-tip symmetric tree and a four-tip asymmetric tree. For both, we assume that the tree topology is fixed and the tip ages in ybp are known.

For the asymmetric tree, the simulation works as follows. A mutation rate is drawn from the prior distribution. The sample dates in expected number of substitutions are calculated. A root age is drawn from the prior. Two node ages are drawn on a uniform distribution between zero and the root age. The times are rank ordered to determine the node ages. If the node ages are younger than the sample dates in a daughter population, the move is rejected. Otherwise, the times are stored. This is repeated until the desired number of samples has been obtained. With a symmetric tree, the simulation works similarly except that the ages for the two (non-root) internal nodes are drawn independently from a uniform distribution between zero and the root age.

### Bayesian Simulation to Validate the Implementation of the MCMC Algorithms

Bayesian simulation is a technique to assess the correctness of a Bayesian inference program, in which a set of parameters of the model are drawn from their prior distributions and then used to simulate a replicate dataset. Then, the inference program is used to analyze each dataset using the priors from which the parameters were drawn, to generate the posterior of the parameters. When the posteriors from replicate datasets are combined, the mixture distribution (or average posterior) should match the prior distribution (Flouri et al., 2022).

Bayesian simulation was conducted on a four-tip symmetric tree with five individuals per species. Sample times were drawn from a uniform distribution between 0 and 50,000 years before present. The sample times were the same for all replicate datasets. Each replicate dataset had 100 loci that were 1000 base pairs in length. Sequence data were simulated with the Jukes–Cantor model (Jukes and Cantor, 1969). As noted above, the prior distribution for some of the parameters in the model is not known analytically. Given the fixed set of sample times and species tree, the rejection simulation method was used to draw parameters from the prior distribution of the τs and μ. The θs were drawn using their analytical prior distributions. We simulated 3000 replicate datasets. The root age was assigned the prior Γ⁢(10,100), the mutation rate had $\mu ∼\Gamma \,\left(10,10^{8}\right)$, and θ∼Γ⁢(8,2000). Full MCMC analysis descriptions are provided in SI Section 2.

### Inference with Extinct Species

#### Simulations: nuclear DNA

**Figure 4:**
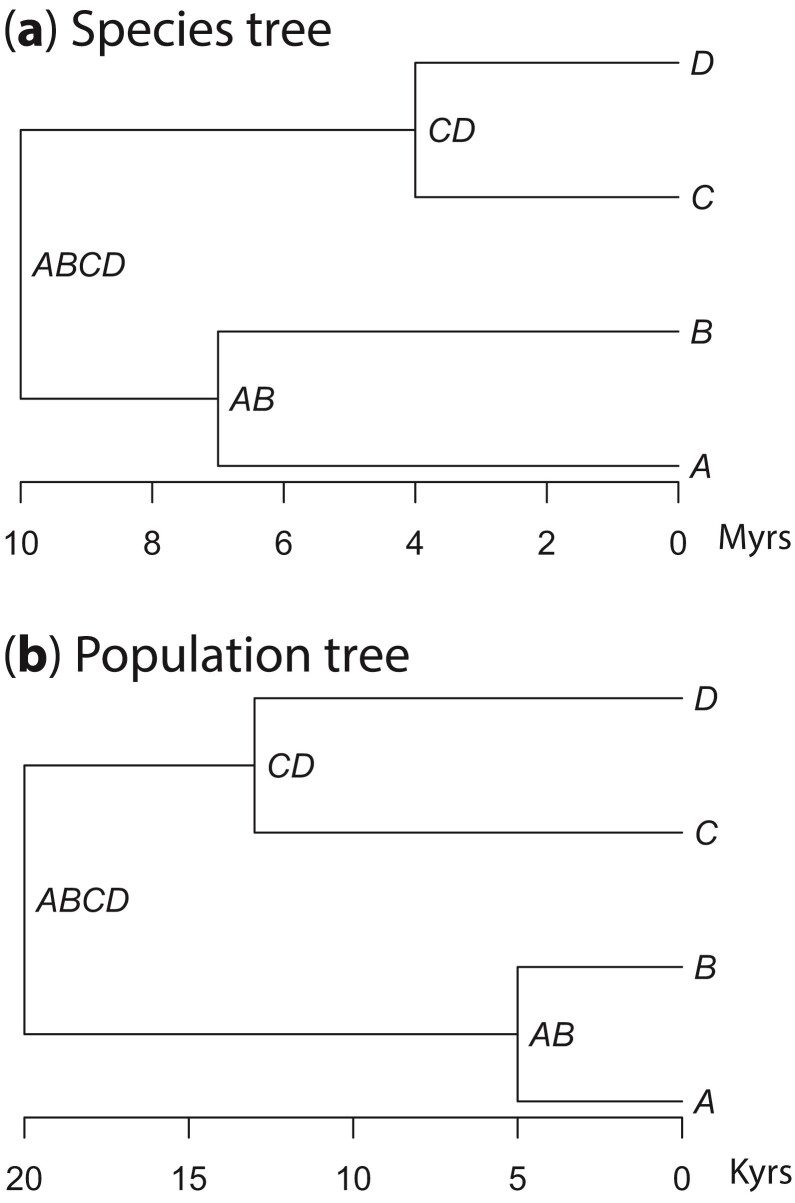
(**a**) The tree used to simulate data with either species A or both A and C extinct. In both cases, the root was 10 million years old, node AB was 7 million years and node CD was 4 millions years old. The extinction occurred at 5000 ybp. (**b**) The tree used to simulate recent population divergences. The root age is 20 kyr. The age of node AB is 5 kyr and the age of node CD is 13 kyr.

To investigate the performance of the method with extinct species, sequence data were simulated for a four-species symmetric tree, with either one or two extinct species (Fig. 4a). We used θ= 0.001 or 0.0001 for all populations, which may be representative of great apes (Kaessmann et al., 2001). For each extant population 3 diploid individuals were sampled, with two phased sequences per locus. For each extinct population either three or six diploid individuals were sampled, with two phased sequences per locus. Datasets had 10, 100, 500, or 2000 loci of 1000 sites each. Sequence data were simulated with a Jukes–Cantor model; for closely related species that experience few multiple substitutions a more complex model is unnecessary. The mutation rate μ was assumed constant across loci with rate $10^{-9}$ mutations per year. For each of the extinct populations, the sample date for each individual was drawn from U⁢(0,1). The extinct populations were assumed to have become extinct 5000 years before present. The date for each individual was rescaled to be between 5000 and 10,000 or 5000 and 50,000 ybp. The number of samples for each extinct species, the number of extinct species, number of loci, value of θ, and age of the samples were examined factorially. For each set of conditions, 20 replicate datasets were simulated. For one replicate, the uniform draws to determine the sampling dates were the same for all of the loci and date ranges. This may mimic the scenario of sampling the same individuals and collecting more loci from them. Relative to the three-individual datasets, three individuals with sampling dates were added in the six-individual datasets. Note that sequence data and coalescent times were simulated independently for each dataset and differ among datasets.

The root age prior was Γ⁢(10,1000). The mutation rate prior was $\mu ∼\Gamma \,\left(10,10^{10}\right)$. The θ prior was $\Gamma \,\left(2,2\times 10^{4}\right)$ and $\Gamma \,\left(2,2\times 10^{5}\right)$ for θ equal to 0.001 and 0.0001, respectively. The priors were chosen to have the means centered at the true parameter values. The mean of the distribution Γ⁢(α,β) is α/β with variance $\alpha /\beta^{2}$.

#### Simulations: mitochondrial DNA

Using the same tree as the nuclear DNA simulations (Fig. 4a), data were simulated with parameters similar to mitochondrial DNA. Specifically, each individual has a single locus that was 16,000 base pairs in length (Boore, 1999) with $\mu =10^{-8}$ substitutions per year. 10 individuals were sampled for each extant population. 10, 20, or 100 individuals were sampled for each extinct population. θ was either 0.0025 or 0.00025 for all populations. θ and the number of individuals sampled in the extinct populations were varied factorially. As in the nuclear datasets, the dates from the 10 individual datasets matched 10 of the individuals in the 20 individual datasets, and the dates from the 20 individual datasets matched 20 of the dates in the 100 individual datasets.

The mutation rate was assigned the prior $\mu ∼\Gamma \,\left(10,10^{9}\right)$. The prior for population sizes was $\theta ∼\Gamma \,\left(2.5,10^{3}\right)$ and $\Gamma \,\left(2.5,10^{4}\right)$ for the larger and smaller values of θ, respectively. The age of the species tree root had the prior τ∼Γ⁢(4,400). Other priors remained the same as in the previous analyses.

### Inference of Recent Population Divergences

To investigate the ability of the method to estimate recent divergence times, data were simulated using a four tip tree with a root age of 20 kyr (Fig. 4b). Three individuals were sampled per population, each with two-phased sequences per locus. Sample ages were drawn between 0 and the divergence time for each population. Datasets were simulated with either 10, 100, 500, or 2000 loci. θ was either 0.001 or 0.0001. The number of replicate datasets simulated for each number of loci was 20. The sample dates were redrawn for each of the 20 replicate datasets.

The root age was assigned the prior $\tau ∼\Gamma \,\left(20,10^{6}\right)$. The mutation rate was assigned the prior $\mu ∼\Gamma \,\left(10,10^{10}\right)$. The prior for θ was $\Gamma \,\left(10,10^{4}\right)$ and $\Gamma \,\left(10,10^{5}\right)$, for the high and low values of θ, respectively. As before, the prior means match the true parameter values. Note that the root age and μ have to be compatible with the fixed sample dates and their effective priors after the truncation differ from the specified gamma distributions.

### Treating Ancient Samples as Contemporary

To examine the effects of ignoring sample dates, the simulated datasets were reanalyzed with all of the sample dates set to zero. The BPP program with tip dating options implemented was also used for these analyses and all priors, including the mutation rate prior, remained thesame.

### MCMC Analysis Details for Simulations

For the simulations with extinct species, a recent population divergence, and ancient samples treated as contemporary, MCMC run length and checks for convergence are described in the SI Section 3. All MCMCs that did not converge were run longer. If they still did not converge, those datasets were excluded from the remaining analysis. At least half of all MCMCs for any particular set of parameters converged. Datasets with more loci were more likely to fail to converge, which is common in phylogenetic analyses.

### Empirical Analysis of Mammoths and Elephants

#### Mitochondrial dataset

The mitochondrial alignment from van der Valk et al. (2021) was downloaded (see Supplementary). This dataset includes forest (*Loxodonta cyclotis*), savanna (*Loxodonta africana*), and Asian (*Elephas maximus*) elephants, woolly mammoths (*Mammuthus primigenius*), Columbian mammoths (*Mammuthus columbi*), and mammoths not identified to the species level. Sequences of unknown age or from unknown species were removed from the dataset. Sequences of Columbian mammoths were also removed, as researchers have suggested a potential hybrid origin (van der Valk et al., 2021). This resulted in 10 elephant sequences and 69 woolly mammoth sequences. The calibrated sample dates published in the original papers were used.

Additional sequences were downloaded from GenBank, including four savanna elephants, eight forest elephants, and three Asian elephants (Supplementary Figure S1). The sequences were realigned with MUSCLE (v3.8.425) using the default settings (Edgar, 2004). Sites in the alignment with more than 25% missing data were removed. This was almost entirely sites at the beginning or end of the alignment. Three sequences from forest elephants were recovered from a ship that sank. The shipwreck year was used as the sample ages for these specimens (Supplementary Figure S1). All other extant species sequences were assigned sample ages of zero.

A HKY+Γ(4) substitution model was used (Hasegawa et al., 1985; Yang, 1994) to account for the extreme transition/transversion rate bias due to DNA degradation. The prior for θ was Γ⁢(2,200). The prior for τ was Γ⁢(22,1000). The prior for μ was $\Gamma \,\left(10,10^{9}\right)$. The reasoning for the prior choices is described in Supplementary material.

#### Nuclear dataset

The dataset from Rohland et al. (2010) was reanalyzed using BPP. The dataset has three extant species: Asian, forest, and savanna elephants; and two extinct species: woolly mammoths and American mastodons (*Mammut americanum*). There are 347 loci, averaging 106 base pairs in length. One individual was sampled per species. The mastodon data are phased, but has one sequence for each individual at each locus, and all other sequences are unphased. The woolly mammoth sample is dated to approximately 43,000 ybp and the mastodon sample is dated to between 50,000 and 130,000 ybp (Römpler et al., 2006; Rohland et al., 2007).

Analyses were conducted using either 50,000, 90,000, or 130,000 ybp as the sample date for the mastodon. The analysis was also repeated without the mastodon sample, both due to the uncertain age and concerns about DNA degradation, as described in original analysis of this dataset (Rohland et al., 2010). The JC model substitution model was used. The prior for τ was Γ⁢(16,1000) and Γ⁢(3.5,1000) with and without the mastodon sample, respectively. The prior for θ was Γ⁢(2,2000) and the prior for μ was $\Gamma \,\left(5,10^{10}\right)$. The reasoning for the prior choices is described in Supplementary material.

## Results

The correctness of the implementation was assessed using Bayesian simulations. The statistical performance of the method was tested using two population histories, a history of ancient species divergence and a recent population divergence, each with four populations. On a four population tree, the method estimates the three divergence times in units of years ($\tau^{△}$) and expected number of substitutions (τ), the seven effective population sizes (θ), and the mutation rate (μ). Simulated nuclear datasets were used for both histories and simulated mitochondrial datasets were used for the species divergence. The effect of treating the aDNA sequences as contemporary was investigated for all datasets. Two elephant and mammoth datasets were analyzed with the new method.

### Bayesian Simulation

**Figure 5:**
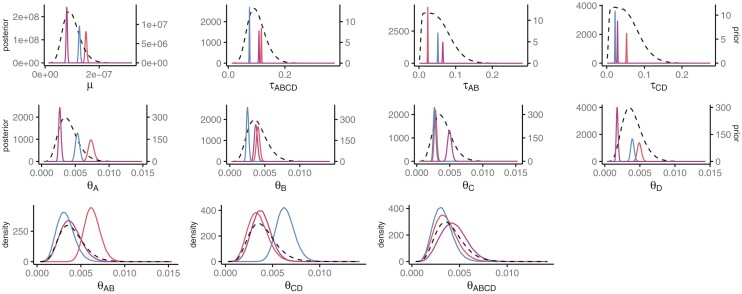
Posterior distributions for individual replicates in the Bayesian simulation. Each solid colored line shows the posterior distributions of representative replicates. The dotted lines show the prior distributions. The y-axis scale is different for the prior and posterior distributions for the first two rows.

**Figure 6:**
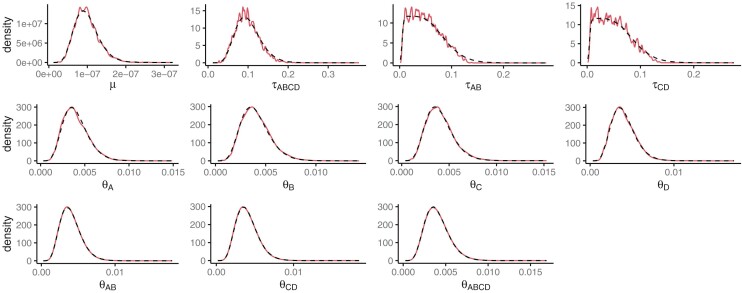
Priors (black-dotted line) and average posterior (solid red line) distributions for parameters in the model in Bayesian simulation.

The data generated for the Bayesian simulations were very informative about the speciation times and the mutation rate (Fig. 5). There was also information about the population sizes in the tip populations. However, there was very little information about the ancestral population sizes, as the posterior distributions very closely resembled the prior distributions. The combined posterior distributions of the MCMCs closely matched the prior distributions for all parameters (Fig. 6). This suggests the program is correctly implemented. For parameters for which the data are more informative, such as the τs (as seen by a low variance in the posterior distributions for individuals replicates), the combined distributions are less smooth as expected.

### Simulations: Species Divergence

#### Inference under the correct model

**Figure 7:**
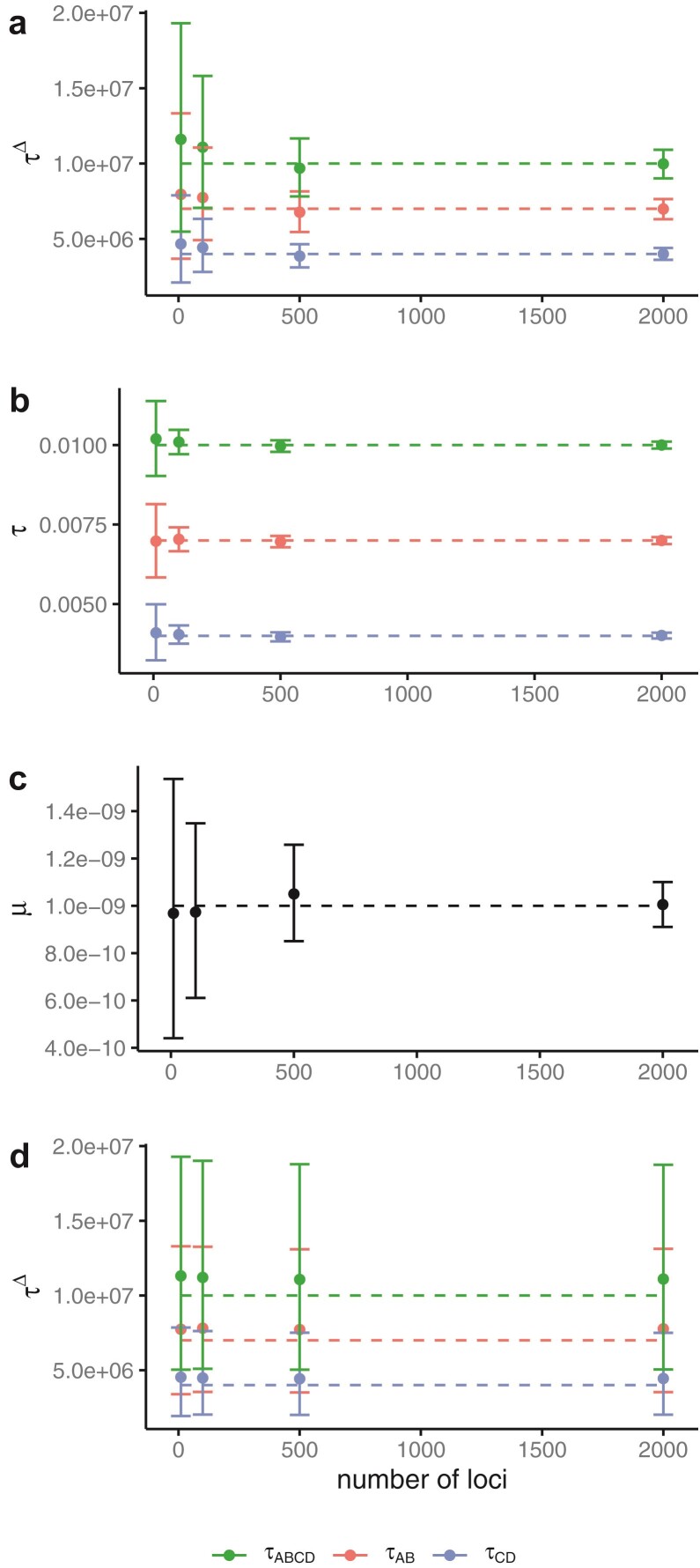
Average posterior means and 95% HPD CIs (bars), over **20** replicate datasets, of (**a**) divergence times in mutations, (**b**) divergence times in years, (**c**) mutation rate, and (**d**) divergence times in years when the samples are treated as contemporary. The data were simulated under the model of fig. 4a with two extinct species (A and C), sample dates are between 5000 and 50,000 years, and θ=0.0001. The dashed lines show the true parameter values.

**Figure 8:**
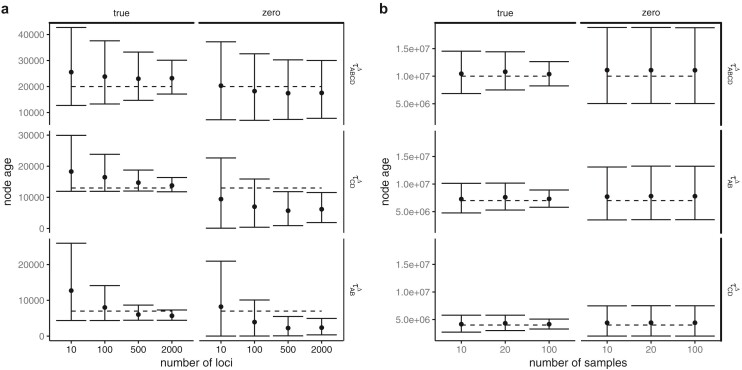
Average posterior means and 95% CIs of divergence times in years over 20 replicate datasets simulated (**a**) under the model of Fig. 4b with recent population divergences and (**b**) under the species tree of Fig. 4a. In each panel, the left column is for results when the sample dates are accommodated in the method, while the right column is for results when all sample dates are set to zero. In both (a) and (b), there are two extinct species (A and C) with sample dates from U(5000, 50,000) ybp and with θ=0.0001. The dashed lines show the true parameter values.

Here we examine the effects of the number of loci and the number of sequences (sampled individuals) on the estimation of mutation rate (μ) and divergence times (τs), obtained from simulated nuclear and mitochondrial sequences. As the number of loci increased with nuclear sequences and the number of samples increased with mitochondrial sequences, estimates of $\tau^{△}$ improved (Figures  7a and 8b). This improvement is a result of better estimates of both μ and τ with more loci (Figure 7b,c). Going from 500 to 2000 loci, the average size of the 95% HPD interval decreases much more for μ than τ. The 95% credible intervals were much smaller for the nuclear analysis with many loci than for the mitochondrial analysis with manyindividuals. The coverages (frequency at which the true parameter value was contained in the 95% credible set) for all datasets with 2000 loci were 97.9% for all divergence times ($\tau_{A\,B\,C\,D}^{△}$, $\tau_{A\,B}^{△}$, $\tau_{C\,D}^{△}$) and 97.6% for μ, respectively. The coverages for all mitochondrial analyses were 97.8%, 97.8%, 97.6%, and 97.6% for $\tau_{A\,B\,C\,D}^{△}$, $\tau_{A\,B}^{△}$, $\tau_{C\,D}^{△}$, and μ, respectively.

**Figure 9:**
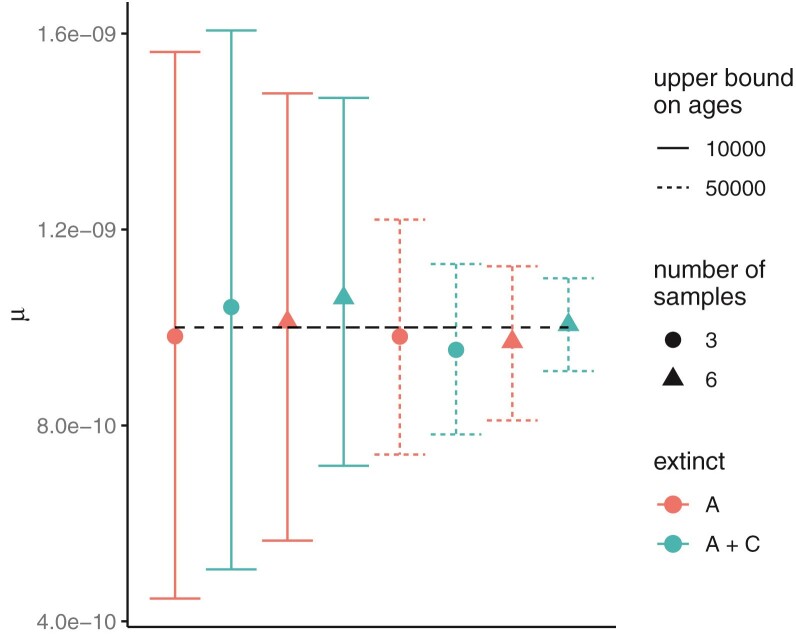
Average posterior means and 95% HPD CIs (bars) of the mutation rate over 20 replicate datasets, each of 2000 loci, simulated under the model of Fig. 4a with θ=0.0001. Solid lines are for sample dates between 5000 to 10,000 ybp while dashed lines are for sample dates between 5000 and 50,000 ybp. Either species A (red) or both A and C (teal) are extinct, and from each extinct species either 3 (circle) or 6 (triangle) samples are taken. The dashed line shows the true parameter value.

The precision and accuracy of estimates of μ in the most informative case (2000 loci) were most impacted by the age range of the samples, with older dates giving more precise estimates (Figure 9, Supplementary Figure S4). Increasing the number of samples for each extinct species and the number of extinct species also improved estimates of μ but to a lesser degree, with the former (number of samples) having the greatest impact. The trends for the estimates of μ are similar with the mitochondrial datasets (Supplementary Figure S4). Using a smaller true value of θ in the simulations for all populations improved estimates of μ and τ (Figure 7, Supplementary Figure S2).

#### Biases when ancient samples were treated as contemporary

Here we examine the potential negative impacts on estimates of μ, τ, and θ if ancient samples are treated as contemporary (e.g., with sample dates set to zero) when analyzing the simulated nuclear sequences. Both μ and $\tau^{△}$ were poorly estimated when ancient samples were treated as contemporary (Figure 7d and Supplementary Fig. S8b) with increased widths of credibility intervals and estimates of θ for extinct species were biased to be too large (Supplementary Figures S3 and S5). Without tip ages, the posterior distribution of μ is the same as the prior distribution because μ and τ are not identifiable in this case—only their product can be estimated. In this case, the estimates of μ are determined solely by the prior and are not impacted by the number of loci (Supplementary Fig. S6).

### Simulations: Population Divergence

#### Inference under the correct model

Here we examine the effects on inference of μ, τ, and θ of increasing the number of loci when considering populations that have recently diverged. There is much less information in this case and priors have more influence on the posterior, even with 2000 loci. As the number of loci increased, estimates of population divergence time (τ) improved, with smaller credible sets and less bias (Fig. 8a). With less data, estimates of τ were upwardly biased, apparently due to the influence of the prior. With 2000 loci, the coverages for $\tau_{A\,B\,C\,D}$, $\tau_{A\,B}$, and $\tau_{C\,D}$ were all 100% and for μ the coverage was 88.9%. The mutation rate was biased downward with smaller amounts of data, likely due to the interaction of the prior and the sample ages. The bias decreased as the amount of data increased (Supplementary Fig. S7). Of the θ parameters, only the root population size was estimated with increased precision as the amount of data increased (Supplementary Fig. S7). This is likely due to the fact that few lineages are expected to coalesce in contemporary populations due to the young divergence times relative to the effective population size (most will coalesce in the root population), so there is little information about contemporary θs.

#### Biases when ancient samples were treated as contemporary

When the samples were treated as contemporary, population divergence times were underestimated (Fig. 8a). This effect was more pronounced for $\tau_{A\,B}$ and $\tau_{C\,D}$ than $\tau_{A\,B\,C\,D}$; the credible sets for these parameters became smaller and the bias became larger as the number of loci increased.

### Analysis of Genomic Data from Elephants and Mammoths

#### Mitochondrial dataset

**Table 2:** Estimates of species divergence times

Analysis	mastodon age	$\tau_{F\,o\,r\,e\,s\,t,S\,a\,v\,a\,n\,n\,a\,h}^{△}$	$\tau_{M\,a\,m\,m\,o\,t\,h,A\,s\,i\,a\,n}^{△}$	$\tau_{M\,a\,m\,m\,o\,t\,h/A\,s\,i\,a\,n,A\,f\,r\,i\,c\,a\,n}^{△}$	$\tau_{M\,a\,t\,s\,t\,o\,d\,o\,n,e\,l\,e\,p\,h\,a\,n\,t/m\,a\,m\,m\,o\,t\,h}^{△}$	$\mu \times 10^{\left(-9\right)}$
mt	NA	29 (6.4 - 49) Ka	1.6 (0.7 - 2.3) Ma	1.9 (1.4 - 2.4) Ma	NA	15 (11 - 18)
nuclear	50 KY	3.0 (0.7 - 6.4) Ma	2.7 (0.7 - 5.7) Ma	5.5 (1.6 - 11.4) Ma	24.7 (7.0 - 50.8) Ma	0.50 (0.12 -0.95)
nuclear	90 KY	3.0 (0.7 - 6.3) Ma	2.7 (0.7 - 5.7) Ma	5.5 (1.6 - 11.3) Ma	24.6 (6.9 - 50.6) Ma	0.50 (0.11 -0.94)
nuclear	130 KY	3.0 (0.7 - 6.3) Ma	2.7 (0.7 - 5.7) Ma	5.5 (1.5 - 11.2) Ma	24.7 (7.1 - 51.0) Ma	0.51 (0.13 -0.95)
nuclear	NA	3.0 (0.8 - 6.4) Ma	2.7 (0.7 - 5.6) Ma	4.8(1.4 - 10.1) Ma	NA	0.51 (0.12 -0.96)
mt (Rohland 2007)	NA	NA	6.7 (5.8-7.7) Ma	7.6 (6.6-8.8) Ma	26 (24-28) Ma	4.2 (3.6 -4.9)
nuclear (Rohland 2010)	NA	(2.6-5.6) Ma	(2.5-5.4) Ma	(4.2 - 9.0) Ma	(34-72) Ma	NA

**Figure 10:**
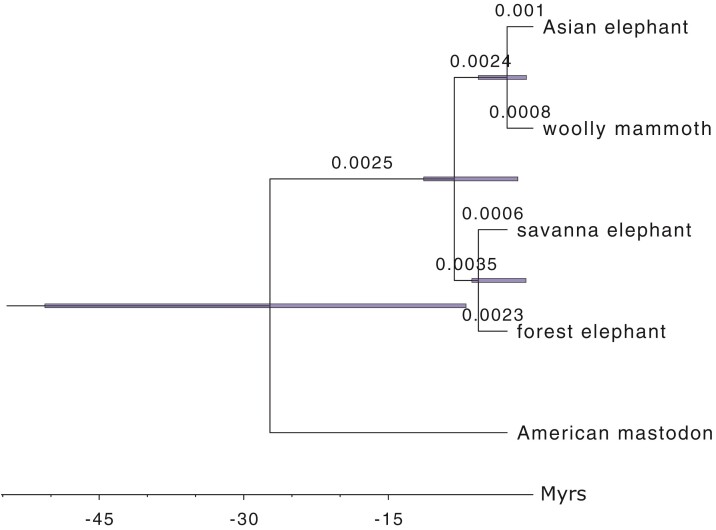
Phylogeny of the elephants and mammoths showing divergence times obtained in BPP analysis of the nuclear data with the mastodon sample date set at 90 KY. Branch lengths represent posterior means while node bars represent the 95% HPD CIs (Table 2).

Estimates of species divergence times (posterior means with the 95% HPD CIs in parentheses) for elephants and mammoths (Fig. 10) in BPP analysis of mitochondrial (mt) and nuclear data. The nuclear data are analyzed assuming different sample date for the mastodon. Results for Rohland (2007) and (2010) are the 95% and 90% CIs, respectively.

The posterior mean divergence time estimate for the two African elephants of 29 Ka was extremely recent and the posterior mean divergence time between the Eurasian and African elephants of 1.6 Ma was much smaller than previous estimates of 7.6 Ma (Table 2). The mean of the posterior distribution of the mutation rate was higher than the mean of the prior. The mean transition transversion ratio, κ, was 46, which is at least an order of magnitude larger than typical empirical datasets for mammals, likely due to DNA degradation.

#### Nuclear dataset

The estimates of the τs and μ were very similar for all analyses, independent of whether the mastodon sample was included in the analysis and of the sample ages used for the mastodon (Table 2). The divergence between the African elephants, Asian elephant and mammoth, African and Eurasian elephants, and mastodon was estimated to be 3.0 (0.7-6.3) Ma, 2.7 (0.6-5.7) Ma, 5.5 (1.6-11.3) Ma, and 24.6 (6.9-50.6) Ma, respectively, for the dating of the mastodon at 90 Ka (Fig. 10). The credible sets were large for $\tau^{△}$, reflecting the limited information about μ available from these data. The estimates were broadly concordant with results from previous studies when analyzing either the nuclear or mitochondrial DNA, though the point estimates of the divergence times tend to be slightly more recent.

## Discussion

Ancient DNA data provide a new way to study historical populations and their relationships to contemporary populations. However, the processes that generate aDNA data do not fit the model assumptions commonly used in aDNA analyses. Here, a new MSC model with tip dating was developed to incorporate the sample ages into population genomic data analysis for multiple species and implemented in BPP.

The simulation study demonstrates that the new method accurately and precisely estimates speciation times in ybp for a variety of data types, including nuclear and mitochondrial sequences, and for population histories with divergence times ranging from several thousand to several million years. In particular, with more loci, more samples, and more extinct species, the confidence intervals for the divergence times become smaller. While the simulation study only used up to 2000 loci, the trend suggests that more loci could lead to even greater improvements in the estimates.

The ability of the method to infer times in years is based on the sampling of genetic data through time. This provides a means to separately estimate the mutation rate and time and thus to convert branch lengths from expected numbers of substitutions to years. Many methods used with aDNA assume a particular mutation rate, which makes the results highly sensitive to that parameter choice. As a Bayesian method, BPP naturally accommodates uncertainty, allowing the prior variance to be chosen to reflect the uncertainty in mutation rate. Our simulation showed that even at the low mutation rate, reliable estimation of the mutation rate and absolute divergence times is possible when a large number of loci are used.

The simulation study also demonstrated detrimental effects that ignoring sample dates can have on inference. In all population histories explored in this simulation study, mutation scaled population sizes (θ) of populations with aDNA were overestimated and divergence time in years had wide credible intervals when ages were ignored. The large credible intervals for divergence times were driven by the uncertainty in mutation rate. Without sample dates, the posterior distribution of μ is the same as the prior distribution, reflecting the lack of separate information about rate and time. For recent population divergences, we observed that the divergence times were underestimated when ancient samples were incorrectly treated as contemporary. This reflects the effects of “missing” mutations between the present time (time zero) and the sample time when using an incorrect model. This effect was not observed for simulations that used extinct species, likely because the missing branch length comprised a much smaller proportion of the branch.

The method assumes that the species tree is known, there is no migration between species, and sequence evolution follows a strict clock. The latest version of BPP relaxes these assumptions (Flouri et al., 2018, 2020, 2023), but does not include tip dating. Future work should merge these models into the program with tip dating. BPP also assumes every sample has a known age, in contrast to programs such as BEAST which allows uncertain ages. Adding unknown sample dates for aDNA to BPP would naturally accommodate the use of data without known sample dates, such as the mastodon data used in this study.

An alternative to tip dating when calibrating a molecular phylogeny is to use fossil calibrations. With aDNA, tip dating can be combined with fossils to estimate a time scaled phylogeny, which is currently possible in BEAST. However, placing fossils on the phylogenetic tree is often difficult and error prone; aDNA samples have the advantage that they can provide calibrations and be positioned on the tree, through the use of sequence data rather than using sparse morphological characters as with fossils. Since fossils provide additional information, a combined approach may allow for more accurate estimation of divergence times, but only if fossils can be accurately placed. BPP does not currently accommodate fossil calibrations. Incorporating fossil calibrations in BPP is another possible area of future work. Fossil calibrations are typically specified in ybp. Future implementations could use branch lengths in ybp to incorporate fossils or different clock models which could allow for simpler algorithms that avoid some of the constraints imposed by using branch length in expected number of substitutions.

The new method had convergence issues, particularly in analysis of large datasets (e.g., with 2000 loci). Ancestral population sizes often did not converge when the rest of the parameters did converge. In a more limited set of simulations, the root age in expected number of substitutions also had convergence issues. Often it is difficult to get large datasets to converge because the likelihood is very concentrated, so the MCMCs mix poorly. This is supported by the fact large datasets converged less often. If the datasets that did not converge had comparatively concentrated likelihoods, discarding those datasets would remove the most informative datasets, thus making the average performance of the method appear worse.

The mitochondrial mammoth and elephant datasets produced younger estimated divergence times, by comparison with previous estimates, when analysed with our new method. The very young divergence time between African elephants may reflect recent migration (reviewed in Roca, 2019). The other divergence times are also younger than the nuclear analysis and other analyses. The estimate of κ, the transition transversion rate ratio, is extremely large, about an order of magnitude higher than typical values. This is likely a result of DNA degradation, which causes excessive post-mortem C to T changes (or G to A changes on the other strand), resulting in very high transition rates. The elevated κ combined with the relatively high mutation rate estimate suggests the dataset contained degraded sequences which inflated mutation rate estimates and resulted in estimation of young divergence times. Research using aDNA, including van der Valk et al. (2021) who generated the dataset we analyzed, typically extensively characterizes evidence for DNA degradation and attempts to remove degraded sequences. However, our results suggest this approach may be insufficient to remove the impact of degradation and highlights the need to systematically assess and potentially model the impact of DNA degradation in downstream analysis (Ho et al., 2007; Axelsson et al., 2008; Rambaut et al., 2009).

The estimates of divergence times with the elephant and mammoth nuclear dataset were broadly consistent with previous estimates using fossil calibrations. The large credible intervals reflect the limited amount of information about μ in the data. The simulation study suggests that more ancient samples and more loci would improve the precision of the estimates of $\tau^{△}$s and μ.

The age of the mastodon sample did not meaningfully impact the results. This may be due to the relatively small number of loci and the short sequence lengths. This suggests that with a limited amount of data, uncertainty in sample dates have less impact on the results than uncertainty in other model parameters. Moreover, existing analyses with small amounts of data with uncertain sample dates may report reasonable results. However, the simulations show that incorrect sample dates negatively affect inference as the amount of data increases. As analyses of large genomic datasets including aDNA become more commonplace, researchers should use methods which explicitly account for sample dates, even with relatively young aDNA.

## Supplementary Material

The mitochondrial alignment and supplemental material is available from the Dryad Digital Repository: https://doi.org/10.5061/dryad.4mw6m90h0.

## Code Availability

A new version of BPP implementing models for dated samples, branched off the original BPP version 4.6, was used for inference. The BPP version used in this manuscript is available at https://github.com/bpp/bpp. Simulation scripts and BPP control files are available at https://github.com/nage0178/tipDating_analysis.

## References

[CIT0001] Angelis K. , Dos Reis M. 2015. The impact of ancestral population size and incomplete lineage sorting on Bayesian estimation of species divergence times. Curr. Zool. 61:874–885.

[CIT0002] Axelsson E. , Willerslev E., Gilbert M.T.P., Nielsen R. 2008. The effect of ancient DNA damage on inferences of demographic histories. Mol. Biol. Evol. 25:2181–2187. 18653730 10.1093/molbev/msn163

[CIT0003] Boore J.L. 1999. Animal mitochondrial genomes. Nucleic Acids Res. 27:1767–1780. 10101183 10.1093/nar/27.8.1767 PMC148383

[CIT0004] Bouckaert R. , Vaughan T.G., Barido-Sottani J., Duchêne S., Fourment M., Gavryushkina A., Heled J., Jones G., Kühnert D., De Maio N., Matschiner M., Mendes F.K., Müller N.F., Ogilvie H.A., du Plessis L., Popinga A., Rambaut A., Rasmussen D., Siveroni I., Suchard M.A., Wu C-H., Xie D., Zhang C., Stadler T., Drummond A.J. 2019. BEAST 2.5: an advanced software platform for Bayesian evolutionary analysis. PLoS Comput. Biol. 15:e1006650. 30958812 10.1371/journal.pcbi.1006650 PMC6472827

[CIT0005] Chang D. , Knapp M., Enk J., Lippold S., Kircher M., Lister A., MacPhee R.D.E., Widga C., Czechowski P., Sommer R., Hodges E., Stümpel N., Barnes I., Dalén L., Derevianko A., Germonpré M., Hillebrand-Voiculescu A., Constantin S., Kuznetsova T., Mol D., Rathgeber T., Rosendahl W., Tikhonov A.N., Willerslev E., Hannon G., Lalueza-Fox C., Joger U., Poinar H., Hofreiter M., Shapiro B. 2017. The evolutionary and phylogeographic history of woolly mammoths: a comprehensive mitogenomic analysis. Sci. Rep. 7:44585. 28327635 10.1038/srep44585 PMC5361112

[CIT0006] Douglas J , Jimenez-Silva CL, Bouckaert R. 2022. StarBeast3: adaptive parallelised Bayesian inference under the multispecies coalescent. Syst. Biol. 71:901–916. 35176772 10.1093/sysbio/syac010 PMC9248896

[CIT0007] Drummond A.J. , Pybus O.G., Rambaut A., Forsberg R., Rodrigo A.G. 2003. Measurably evolving populations. Trends Ecol. Evol. 18:481–488.

[CIT0008] Edgar R.C. 2004. MUSCLE: multiple sequence alignment with high accuracy and high throughput. Nucleic Acids Res. 32:1792–1797. 15034147 10.1093/nar/gkh340 PMC390337

[CIT0009] Felsenstein J. 1981. Evolutionary trees from DNA sequences: a maximum likelihood approach. J. Mol. Evol. 17:368–376. 7288891 10.1007/BF01734359

[CIT0010] Flouri T. , Huang J., Jiao X., Kapli P., Rannala B., Yang Z. 2022. Bayesian phylogenetic inference using relaxed-clocks and the multispecies coalescent. Mol. Biol. and Evol. 39:msac161. 35907248 10.1093/molbev/msac161 PMC9366188

[CIT0011] Flouri T. , Jiao X., Huang J., Rannala B., Yang Z. 2023. Efficient Bayesian inference under the multispecies coalescent with migration. Proc. Natl. Acad. Sci. USA. 120:e2310708120. 37871206 10.1073/pnas.2310708120 PMC10622872

[CIT0012] Flouri T. , Jiao X., Rannala B., Yang Z. 2018. Species tree inference with BPP using genomic sequences and the multispecies coalescent. Mol. Biol. and Evol. 35:2585–2593. 30053098 10.1093/molbev/msy147 PMC6188564

[CIT0013] Flouri T. , Jiao X., Rannala B., Yang Z. 2020. A Bayesian implementation of the multispecies coalescent model with introgression for phylogenomic analysis. Mol. Biol. Evol. 37:1211–1223. 31825513 10.1093/molbev/msz296 PMC7086182

[CIT0014] Fortes G.G. , Grandal-d’Anglade A., Kolbe B., Fernandes D., Meleg I.N., García-Vázquez A., Pinto-Llona A.C., Constantin S., de Torres T.J., Ortiz J.E., Frischauf C., Rabeder G., Hofreiter M., Barlow A. 2016. Ancient DNA reveals differences in behaviour and sociality between brown bears and extinct cave bears. Mol. Ecol. 25:4907–4918. 27506329 10.1111/mec.13800

[CIT0015] Gillespie J.H. , Langley C.H. 1979. Are evolutionary rates really variable? J. Mol. Evol. 13:27–34. 458870 10.1007/BF01732751

[CIT0016] Green R.E. , Krause J., Briggs A.W., Maricic T., Stenzel U., Kircher M., Patterson N., Li H., Zhai W., Fritz M.H., Hansen N.F., Durand E.Y., Malaspinas A.S., Jensen J.D., Marques-Bonet T., Alkan C., Prufer K., Meyer M., Burbano H.A., Good J.M., Schultz R., Aximu-Petri A., Butthof A., Hober B., Hoffner B., Siegemund M., Weihmann A., Nusbaum C., Lander E.S., Russ C., Novod N., Affourtit J., Egholm M., Verna C., Rudan P., Brajkovic D., Kucan Z., Gusic I., Doronichev V.B., Golovanova L.V., Lalueza-Fox C., de la Rasilla M., Fortea J., Rosas A., Schmitz R.W., Johnson P.L., Eichler E.E., Falush D., Birney E., Mullikin J.C., Slatkin M., Nielsen R., Kelso J., Lachmann M., Reich D., Paabo S. 2010. A draft sequence of the neandertal genome. Science. 328:710–722. 20448178 10.1126/science.1188021 PMC5100745

[CIT0017] Hasegawa M. , Kishino H., Yano T. 1985. Dating of the human-ape splitting by a molecular clock of mitochondrial DNA. J. Mol. Evol. 22:160–174. 3934395 10.1007/BF02101694

[CIT0018] Ho S.Y.W. , Heupink T.H., Rambaut A., Shapiro B. 2007. Bayesian estimation of sequence damage in ancient DNA. Mol. Biol. Evol. 24:1416–1422. 17395598 10.1093/molbev/msm062

[CIT0019] Horsburgh K.A. , Gosling A.L., Cochrane E.E., Kirch P.V., Swift J.A., McCoy M.D. 2022. Origins of Polynesian pigs revealed by mitochondrial whole genome ancient DNA. Animals. 12:2469. 36139328 10.3390/ani12182469 PMC9495175

[CIT0020] Jukes T. , Cantor C. 1969. Evolution of protein molecules. New York: Academic Press. p. 21–123.

[CIT0021] Kaessmann H. , Wiebe V., Weiss G., Pääbo S. 2001. Great ape DNA sequences reveal a reduced diversity and an expansion in humans. Nat Genet. 27:155–156. 11175781 10.1038/84773

[CIT0022] Li H. , Durbin R. 2011. Inference of human population history from individual whole-genome sequences. Nature. 475:493–496. 21753753 10.1038/nature10231 PMC3154645

[CIT0023] Li W.H. , Tanimura M., Sharp P.M. 1988. Rates and dates of divergence between AIDS virus nucleotide sequences. Mol. Biol. Evol. 5:313–330. 3405075 10.1093/oxfordjournals.molbev.a040503

[CIT0024] Lord E. , Dussex N., Kierczak M., Díez-del Molino D., Ryder O.A., Stanton D.W.G., Gilbert M.T.P., Sánchez-Barreiro F., Zhang G., Sinding M.H.S., Lorenzen E.D., Willerslev E., Protopopov A., Shidlovskiy F., Fedorov S., Bocherens H., Nathan S.K.S.S., Goossens B., van der Plicht J., Chan Y.L., Prost S., Potapova O., Kirillova I., Lister A.M., Heintzman P.D., Kapp J.D., Shapiro B., Vartanyan S., Götherström A., Dalén L. 2020. Pre-extinction demographic stability and genomic signatures of adaptation in the woolly rhinoceros. Curr. Biol. 30:3871–3879.e7. 32795436 10.1016/j.cub.2020.07.046

[CIT0025] Mailund T. , Halager A.E., Westergaard M., Dutheil J.Y., Munch K., Andersen L.N., Lunter G., Prüfer K., Scally A., Hobolth A., Schierup M.H. 2012. A new isolation with migration model along complete genomes infers very different divergence processes among closely related great ape species. PLoS Genet. 8:e1003125. 23284294 10.1371/journal.pgen.1003125 PMC3527290

[CIT0026] Miller W. , Schuster S.C., Welch A.J., Ratan A., Bedoya-Reina O.C., Zhao F., Kim H.L., Burhans R.C., Drautz D.I., Wittekindt N.E., Tomsho L.P., Ibarra-Laclette E., Herrera-Estrella L., Peacock E., Farley S., Sage G.K., Rode K., Obbard M., Montiel R., Bachmann L., Ingólfsson Ó., Aars J., Mailund T., Wiig Ø., Talbot S.L., Lindqvist C. 2012. Polar and brown bear genomes reveal ancient admixture and demographic footprints of past climate change. Proc. Natl. Acad. Sci. USA. 109:E2382–E2390. 22826254 10.1073/pnas.1210506109 PMC3437856

[CIT0027] Nielsen R. , Akey J.M., Jakobsson M., Pritchard J.K., Tishkoff S., Willerslev E. 2017. Tracing the peopling of the world through genomics. Nature. 541:302–310. 28102248 10.1038/nature21347 PMC5772775

[CIT0028] Orlando L. , Allaby R., Skoglund P., Der Sarkissian C., Stockhammer P.W., Ávila Arcos M.C., Fu Q., Krause J., Willerslev E., Stone A.C., Warinner C. 2021. Ancient DNA analysis. Nat. Rev. Methods Primers. 1:1–26.

[CIT0029] Palkopoulou E. , Lipson M., Mallick S,. Nielsen S., Rohland N., Baleka S., Karpinski E., Ivancevic A.M., To T.H., Kortschak R.D., Raison J.M., Qu Z., Chin T.J., Alt K.W., Claesson S., Dalén L., MacPhee R.D.E., Meller H., Roca A.L., Ryder O.A., Heiman D., Young S., Breen M., Williams C., Aken B.L., Ruffier M., Karlsson E., Johnson J., Di Palma F., Alfoldi J., Adelson D.L., Mailund T., Munch K., Lindblad-Toh K., Hofreiter M., Poinar H., Reich D. 2018. A comprehensive genomic history of extinct and living elephants. Proc. Natl. Acad. Sci. USA. 115:E2566–E2574. 29483247 10.1073/pnas.1720554115 PMC5856550

[CIT0030] Palkopoulou E , Mallick S, Skoglund P, Enk J, Rohland N., Li H., Omrak A., Vartanyan S., Poinar H., Götherström A., Reich D., Dalén L. 2015. Complete genomes reveal signatures of demographic and genetic declines in the woolly mammoth. Curr. Biol. 25:1395–1400. 25913407 10.1016/j.cub.2015.04.007 PMC4439331

[CIT0031] Rambaut A. 2000. Estimating the rate of molecular evolution: incorporating non-contemporaneous sequences into maximum likelihood phylogenies. Bioinformatics. 16:395–399. 10869038 10.1093/bioinformatics/16.4.395

[CIT0032] Rambaut A. , Ho S.Y., Drummond A.J., Shapiro B. 2009. Accommodating the effect of ancient DNA damage on inferences of demographic histories. Mol. Biol. Evol. 26:245–248. 19001634 10.1093/molbev/msn256

[CIT0033] Ramos-Madrigal J. , Smith B.D., Moreno-Mayar J.V., Gopalakrishnan S., Ross-Ibarra J., Gilbert M.T.P., Wales N. 2016. Genome sequence of a 5,310-year-old maize cob provides insights into the early stages of maize domestication. Curr. Biol. 26:3195–3201. 27866890 10.1016/j.cub.2016.09.036

[CIT0034] Rannala B. 2016. Conceptual issues in Bayesian divergence time estimation. Philos. Trans. R. Soc. Lond. B Biol. Sci. 371:20150134. 27325831 10.1098/rstb.2015.0134 PMC4920335

[CIT0035] Rannala B. , Yang Z. 2003. Bayes estimation of species divergence times and ancestral population sizes using DNA sequences from multiple loci. Genetics. 164:1645–1656. 12930768 10.1093/genetics/164.4.1645 PMC1462670

[CIT0036] Rannala B. , Yang Z. 2017. Efficient Bayesian species tree inference under the multispecies coalescent. Syst. Biol. 66:823–842. 28053140 10.1093/sysbio/syw119 PMC8562347

[CIT0037] Rasmussen M. , Li Y., Lindgreen S., Pedersen J.S., Albrechtsen A., Moltke I., Metspalu M., Metspalu E., Kivisild T., Gupta R., Bertalan M., Nielsen K., Gilbert M.T.P., Wang Y., Raghavan M., Campos P.F., Kamp H.M., Wilson A.S., Gledhill A., Tridico S., Bunce M., Lorenzen E.D., Binladen J., Guo X., Zhao J., Zhang X., Zhang H., Li Z., Chen M., Orlando L., Kristiansen K., Bak M., Tommerup N., Bendixen C., Pierre T.L., Grønnow B., Meldgaard M., Andreasen C., Fedorova S. A, Osipova L.P., Higham T.F.G., Ramsey C.B., Hansen T.V.O., Nielsen F.C., Crawford M.H., Brunak S., Sicheritz-Pontén T., Villems R., Nielsen R., Krogh A., Wang J., Willerslev E. 2010. Ancient human genome sequence of an extinct palaeo-eskimo. Nature. 463:757–762. 20148029 10.1038/nature08835 PMC3951495

[CIT0038] Roca A.L. 2019. African elephant genetics: enigmas and anomalies. J. Genet. 98:83. 31544772

[CIT0039] Rodrigo A.G. , Felsenstein J. 1999. Coalescent approaches to HIV population genetics. In: K.A. Crandall, editor. The evolution of HIV. Baltimore: Johns Hopkins University Press. p. 233–272.

[CIT0040] Rohland N. , Malaspinas A.S., Pollack J.L., Slatkin M., Matheus P., Hofreiter M. 2007. Proboscidean mitogenomics: chronology and mode of elephant evolution using mastodon as outgroup. PLoS Biol. 5:e207. 17676977 10.1371/journal.pbio.0050207 PMC1925134

[CIT0041] Rohland N. , Reich D., Mallick S., Meyer M., Green R.E., Georgiadis N.J., Roca A.L., Hofreiter M. 2010. Genomic DNA sequences from mastodon and woolly mammoth reveal deep speciation of forest and savanna elephants. PLoS Biol. 8:e1000564. 21203580 10.1371/journal.pbio.1000564 PMC3006346

[CIT0042] Römpler H. , Rohland N., Lalueza-Fox C., Willerslev E., Kuznetsova T., Rabeder G., Bertranpetit J., Schöneberg T., Hofreiter M. 2006. Nuclear gene indicates coat-color polymorphism in mammoths. Science. 313:62–62. 16825562 10.1126/science.1128994

[CIT0043] Soubrier J. , Gower G., Chen K., Richards S.M., Llamas B., Mitchell K.J., Ho S.Y.W., Kosintsev P., Lee M.S.Y., Baryshnikov G., Bollongino R., Bover P., Burger J., Chivall D., Crégut-Bonnoure E., Decker J.E., Doronichev V.B., Douka K., Fordham D.A., Fontana F., Fritz C., Glimmerveen J., Golovanova L.V., Groves C., Guerreschi A., Haak W., Higham T., Hofman-Kamińska E., Immel A., Julien M.A., Krause J., Krotova O., Langbein F., Larson G., Rohrlach A., Scheu A., Schnabel R.D., Taylor J.F., Tokarska M., Tosello G., van der Plicht J., van Loenen A., Vigne J.D., Wooley O., Orlando L., Kowalczyk R., Shapiro B., Cooper A. 2016. Early cave art and ancient DNA record the origin of European bison. Nat. Commun. 7:13158. 27754477 10.1038/ncomms13158 PMC5071849

[CIT0044] Suchard M.A. , Lemey P., Baele G., Ayres D.L., Drummond A.J., Rambaut A. 2018. Bayesian phylogenetic and phylodynamic data integration using BEAST 1.10. Virus Evol. 4:vey016. 29942656 10.1093/ve/vey016 PMC6007674

[CIT0045] Takahata N. , Satta Y., Klein J. 1995. Divergence time and population size in the lineage leading to modern humans. Theor. Popul. Biol. 48:198–221. 7482371 10.1006/tpbi.1995.1026

[CIT0046] Thorne J.L. , Kishino H., Painter I.S. 1998. Estimating the rate of evolution of the rate of molecular evolution. Mol. Biol. Evol. 15:1647–1657. 9866200 10.1093/oxfordjournals.molbev.a025892

[CIT0047] van der Valk T. , Pečnerová P., Díez-del Molino D., Bergström A., Oppenheimer J., Hartmann S., Xenikoudakis G., Thomas J.A., Dehasque M., Sağlıcan E., Fidan F.R., Barnes I., Liu S., Somel M., Heintzman P.D., Nikolskiy P., Shapiro B., Skoglund P., Hofreiter M., Lister A.M., Götherström A., Dalén L. 2021. Million-year-old DNA sheds light on the genomic history of mammoths. Nature. 591:265–269. 33597750 10.1038/s41586-021-03224-9 PMC7116897

[CIT0048] Yang Z. 1994. Maximum likelihood phylogenetic estimation from DNA sequences with variable rates over sites: approximate methods. J. Mol. Evol. 39:306–314. 7932792 10.1007/BF00160154

[CIT0049] Yang Z. 2014. Molecular evolution: a statistical approach. Oxford: Oxford University Press.

[CIT0050] Yang Z. , Rodríguez C.E. 2013. Searching for efficient Markov chain Monte Carlo proposal kernels. Proc. Natl. Acad. Sci. USA. 110:19307–19312. 24218600 10.1073/pnas.1311790110 PMC3845170

